# Development and internal validation of a prediction model for suboptimal bowel preparation in individuals aged ≤50 years undergoing colonoscopy

**DOI:** 10.3389/fmed.2026.1939670

**Published:** 2026-09-23

**Authors:** Fengjun Jiang, Xiaoting Chen, Zehui Wei, Shanshan Lin, Lexin Liu, Pengli Ma, Yinxia Xiao, Zhaoli Fu

**Affiliations:** 1 Digestive Endoscopy Department, Guangdong Provincial Hospital of Chinese Medicine, Guangzhou, Guangdong, China; 2 Digestive Endoscopy Department, Shenzhen Hospital (Fu Tian) of Guangzhou University of Chinese Medicine, Shenzhen, Guangdong, China; 3 Hepatology Department, Guangdong Provincial Hospital of Chinese Medicine, Guangzhou, Guangdong, China

**Keywords:** Boston Bowel Preparation Scale, bowel preparation, colonoscopy, early-onset colorectal cancer, prediction model, young adults

## Abstract

**Background:**

The incidence of early-onset colorectal cancer (EOCRC) has increased in recent decades, leading to more frequent colonoscopy use in younger adults. Adequate bowel preparation is essential for colonoscopy quality, but predictors of suboptimal bowel preparation and individualized risk assessment tools remain insufficiently defined in this population.

**Methods:**

We conducted a retrospective cohort study of 2,283 consecutive individuals aged ≤50 years who underwent complete colonoscopy between June 2017 and December 2025. Bowel preparation quality was assessed using the Boston Bowel Preparation Scale (BBPS). Suboptimal bowel preparation was defined as a total BBPS score <6 or any segmental score <2. Colonoscopy indication was evaluated separately. Two multivariable logistic regression models were defined according to predictor availability relative to selection of the bowel-preparation regimen: a baseline model containing patient characteristics available before selection of the bowel-preparation regimen and an extended model that additionally included preparation and treatment variables. Performance was assessed using receiver operating characteristic analysis, 1,000-resample bootstrap internal validation, calibration, Brier score, and decision curve analysis (DCA). Exploratory analyses examined age-group-by-predictor interactions in a same-centre older comparison cohort, calendar-period adjustment, and internal temporal validation.

**Results:**

Suboptimal bowel preparation occurred in 426 participants (18.7%). Colonoscopy indication was not associated with suboptimal preparation after adjustment (global likelihood-ratio *P* = 0.358). In the extended model, diabetes mellitus (OR: 10.701, 95% CI: 7.312–15.660) and constipation (OR: 6.250, 95% CI: 4.619–8.458) were strongly associated with suboptimal preparation. Split-dose preparation (OR: 0.459, 95% CI: 0.362–0.583) and linaclotide use (OR: 0.375, 95% CI: 0.196–0.718) were associated with lower odds. Apparent AUCs were 0.722 for the baseline model and 0.761 for the extended model; corresponding optimism-corrected AUCs were 0.714 and 0.752. The extended model improved discrimination modestly (ΔAUC = 0.039; likelihood-ratio *P* < 0.001). In internal temporal validation (development, 2017–2022; validation, 2023–2025), the extended model had an AUC of 0.769 but underestimated later risk (calibration intercept 0.389; slope 0.830).

**Conclusion:**

Patient characteristics available before regimen selection provided moderate discrimination for suboptimal bowel preparation. Adding preparation and treatment variables modestly improved performance and estimated residual risk after the selected regimen. Because these variables were not randomly assigned, their associations should not be interpreted as causal treatment effects. The model was developed for individuals aged ≤50 years who completed standard preparation without rescue cleansing and underwent complete colonoscopy. Its absolute risk estimates reflect the pooled 2017–2025 case mix; recalibration or model updating and external validation are required before clinical use.

## 1 Introduction

Globally, colorectal cancer (CRC) is the third most commonly diagnosed malignancy and the second leading cause of cancer-related death (1). In China, CRC remains a major public health challenge, ranking among the most frequently diagnosed malignancies and contributing substantially to cancer-related mortality (2). Over the past two decades, CRC incidence has stabilized or declined in many older populations. In contrast, early-onset colorectal cancer (EOCRC) has increased, raising concerns about prevention and early detection in younger adults (3, 4). Because most CRCs arise through the adenoma–carcinoma sequence, colonoscopy is central to the detection and removal of precancerous lesions (5). Reflecting the increasing burden of EOCRC, the United States Preventive Services Task Force lowered the recommended starting age for average-risk CRC screening from 50 to 45 years in 2021 (6).

Colonoscopy is a key modality for CRC screening because it allows direct visualization of the colonic mucosa and removal of neoplastic lesions during the same procedure (7). Its diagnostic performance, however, depends strongly on bowel preparation quality. Inadequate bowel cleansing has been associated with longer procedure time, lower adenoma detection, higher miss rates of advanced lesions, and earlier repeat examinations (8).

Previous studies have identified several patient-related predictors of inadequate bowel preparation, including male sex, older age, inpatient status, hypertension, diabetes mellitus, constipation, and liver cirrhosis (9, 10). Procedure-related factors, such as anesthesia or sedation use, bowel preparation agent, preparation timing, and adherence to preparation instructions, have also been associated with bowel cleansing quality (10, 11). More recently, adjunctive agents such as linaclotide have been evaluated as strategies to improve bowel preparation, particularly in patients with constipation (12, 13). However, most available evidence has been derived from mixed-age or older populations, and data focused on younger adults remain limited.

With the expanding use of colonoscopy in younger adults, determinants of suboptimal bowel preparation in this population need to be better defined. Evidence focused specifically on individuals aged ≤50 years remains limited, and prediction tools tailored to this age group are lacking. Therefore, this study aimed to identify predictors of suboptimal bowel preparation in individuals aged ≤50 years, develop and internally validate a multivariable prediction model, and examine whether predictor associations differed across colonic segments.

## 2 Materials and methods

### 2.1 Study design and population

This retrospective cohort study aimed to develop and internally validate a multivariable prediction model for identifying suboptimal bowel preparation in individuals aged ≤50 years undergoing colonoscopy. The study was conducted at The Sixth Affiliated Hospital of Guangzhou University of Chinese Medicine, a tertiary referral center providing gastrointestinal endoscopic services.

A total of 10,185 colonoscopy examinations were assessed. Before formation of the initial analytic cohort, 380 examinations requiring rescue cleansing were excluded (103 in individuals aged ≤50 years and 277 in those aged >50 years), leaving 9,805 examinations. The 380 rescue-cleansing examinations and the subsequent 304 incomplete examinations were counted as mutually exclusive groups: examinations were first excluded for rescue cleansing, and incomplete colonoscopy was then assessed among the remaining 9,805 examinations. Some examinations in the rescue group remained incomplete after rescue, but these were not counted again among the subsequent 304 examinations. Complete colonoscopy was defined as successful advancement of the colonoscope proximal to the ileocecal valve and fully into the cecal caput, or into the terminal ileum. Incomplete colonoscopy occurred in 304 examinations because of poor bowel preparation (*n* = 137; 55 aged ≤50 years and 82 aged >50 years), pain or inadequate sedation (*n* = 52), tumour, stenosis, or obstruction (*n* = 49), looping or a tortuous colon (*n* = 39), or other reasons (*n* = 27), leaving 9,501 complete colonoscopies. We then excluded 235 examinations involving regular or long-term mosapride use before the bowel-preparation period and 214 involving regular or long-term linaclotide use before that period, leaving an eligible cohort of 9,052 examinations. This cohort comprised 2,765 individuals aged ≤50 years and 6,287 aged >50 years. Among the younger group, a history of CRC (*n* = 253), IBD (*n* = 216), or FAP (*n* = 13) was excluded, yielding the primary cohort of 2,283 individuals. The older comparison cohort was formed after the additional exclusions shown in Figure 1. For the outcome-related sensitivity analysis in the younger group, 13 of the 55 examinations that were incomplete because of poor preparation were excluded because of concomitant colorectal cancer or tumour (*n* = 8), IBD (*n* = 1), or regular or long-term mosapride or linaclotide use before the bowel-preparation period (*n* = 4), leaving 42 otherwise eligible examinations. Of the 103 younger examinations requiring rescue cleansing, 13 were excluded because of CRC (*n* = 4), IBD (*n* = 1), incomplete cecal intubation after rescue (*n* = 2), or regular or long-term mosapride or linaclotide use before the bowel-preparation period (*n* = 6), leaving 90 otherwise eligible examinations.

**Figure 1:**
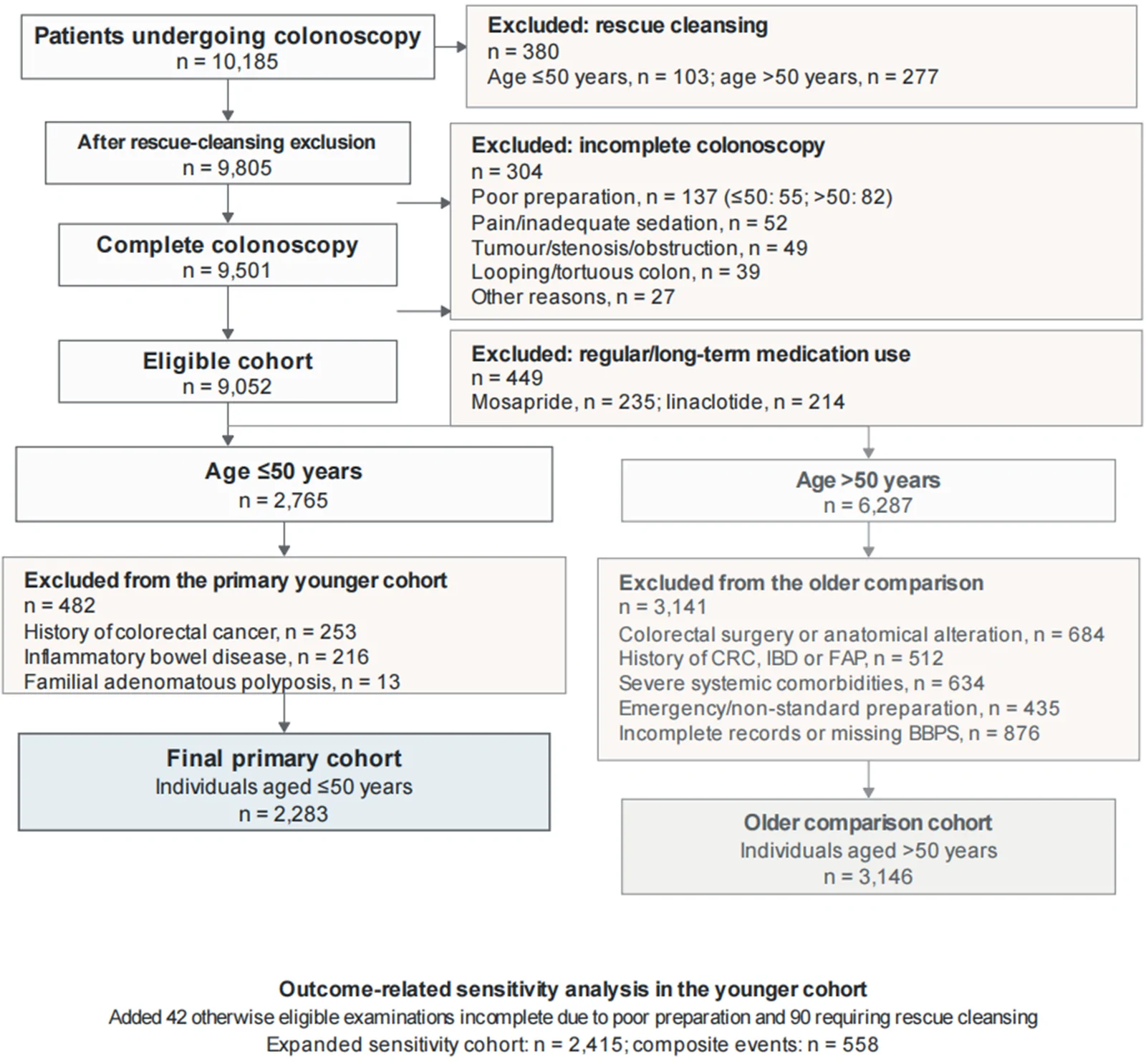
Flow diagram for cohort formation. Among 10,185 examinations assessed, 380 requiring rescue cleansing were excluded (103 in individuals aged ≤50 years and 277 in those aged >50 years), leaving 9,805 examinations. A further 304 incomplete colonoscopies were excluded, including 137 incomplete because of poor bowel preparation (55 aged ≤50 years and 82 aged >50 years), leaving 9,501 complete examinations. Regular or long-term use of mosapride or linaclotide before the bowel-preparation period was then excluded, leaving 9,052 eligible examinations. The younger primary cohort and older comparison cohort were formed as shown. CRC, colorectal cancer; IBD, inflammatory bowel disease; FAP, familial adenomatous polyposis; BBPS, Boston bowel preparation scale.

The study protocol was approved by the Ethics Committee of the Sixth Affiliated Hospital of Guangzhou University of Chinese Medicine [also known as Shenzhen Hospital (Fu Tian) of Guangzhou University of Chinese Medicine], Shenzhen, China [approval number: GZYLL(KY)-2022-032]. Given the retrospective nature of the study, the requirement for written informed consent was waived. The study was conducted in accordance with the principles of the Declaration of Helsinki. Missing values were *n* = 0 for each predictor in the baseline and extended models: age, sex, inpatient status, previous colonoscopy, family history of gastrointestinal malignancy, hypertension, diabetes mellitus, constipation, mosapride use, preparation timing, preparation agent, and linaclotide use. Anaesthesia and colonoscopy indication, which were used only in secondary analyses, also had no missing values. The primary analyses therefore used complete cases.

### 2.2 Clinical data collection and candidate predictors

Demographic, clinical, and procedure-related data were extracted from the electronic medical record system and endoscopy reporting database. Patient characteristics included age, sex, inpatient status, family history of gastrointestinal malignancy, previous colonoscopy, diabetes mellitus, hypertension, constipation, and colonoscopy indication (9, 12–14). Diabetes mellitus was defined by a documented previous diagnosis or current use of glucose-lowering medication. Constipation was defined by a clinical diagnosis recorded in the medical record. Colonoscopy indication was coded as polyp surveillance, family history, symptomatic investigation, or screening. Preparation-related variables comprised anaesthesia or sedation, mosapride, linaclotide, bowel-preparation agent, and same-day vs. split-dose administration.

Two bowel-preparation agents were used: polyethylene glycol-electrolyte solution (PEG-ES; Shenzhen Wanhe Pharmaceutical Co., Ltd., Shenzhen, China) and 20% mannitol (Shijiazhuang No. 4 Pharmaceutical Co., Ltd., Shijiazhuang, China). Outpatients generally received either agent, whereas inpatients generally received PEG-ES. No other prespecified patient-level criteria determined agent selection. Same-day or split-dose administration was selected according to the scheduled colonoscopy time and the clinical bowel-preparation protocol.

For PEG-ES, each sachet was dissolved in 1 L water. The same-day regimen comprised 3 L beginning approximately 4–6 h before colonoscopy, administered at approximately 250 mL every 10–15 min. For split dosing, 1 L was taken at approximately 21:00 on the preceding evening and the remaining 2 L was taken in two portions beginning approximately 4–6 h before colonoscopy.

For mannitol, the total dose was 500 mL of 20% solution with 2 L additional warm water. In the same-day regimen, 125, 125, and 250 mL mannitol were followed by 500, 500, and 1,000 mL water, respectively, beginning approximately 4–6 h before colonoscopy. In the split-dose regimen, 125 mL mannitol plus 500 mL water was taken on the preceding evening; the remaining 375 mL mannitol (125 and 250 mL) plus 1,500 mL water was taken in two portions beginning approximately 4–6 h before colonoscopy.

Linaclotide and mosapride were used as adjuncts during the bowel-preparation period rather than as regular long-term constipation therapy. Their use was not determined by a written risk-stratification protocol. The treating physician selected an adjunct according to clinical judgement and anticipated preparation difficulty, potentially considering constipation, previous difficult or failed preparation, and other clinical indicators of difficult cleansing. These considerations were not prospectively recorded as formal prescribing criteria. Linaclotide was administered at 290 μg once daily beginning 1–3 days before colonoscopy, with the starting day selected by the treating physician. Mosapride 5 mg was administered 30 min before each dose of bowel-cleansing agent, for three doses. The two adjuncts were generally not used together.

No other adjunctive laxatives were included in the routine protocol. Rescue cleansing, when clinically required, consisted of three glycerol enemas (total 60 mL) plus a 500 mL saline cleansing enema. Overall, 380 examinations required rescue cleansing: 103 in individuals aged ≤50 years and 277 in those aged >50 years. These examinations were excluded before formation of the cohort of 9,805 examinations.

All patients received standardized bowel preparation instructions before colonoscopy. They were advised to avoid high-fiber foods for three days before the examination and to consume a clear-liquid diet on the day preceding the procedure. Trained nurses provided standardized education regarding the bowel preparation agent and administration schedule. Written materials, visual illustrations, and instructional videos were used to support patient understanding. Data extraction was independently verified by two investigators to ensure data accuracy.

### 2.3 Assessment of bowel preparation quality

The primary outcome was suboptimal bowel preparation. Bowel preparation quality was assessed using the Boston Bowel Preparation Scale (BBPS), a validated instrument for evaluating bowel cleanliness during colonoscopy (15, 16). BBPS scores were assigned immediately after the examination by experienced endoscopists who routinely performed colonoscopic quality assessment. Because scoring was performed as part of routine clinical care before the present retrospective analysis, no study-specific assessor-blinding procedure was implemented; the endoscopists had access to the clinical information available at the time of colonoscopy.

The BBPS evaluates three colonic segments: the right colon, transverse colon, and left colon. Each segment is scored from 0 to 3, yielding a total score from 0 to 9, with higher scores indicating better bowel preparation.

Suboptimal bowel preparation was defined as a total BBPS score <6 or any segmental BBPS score <2, in accordance with established criteria (14, 17). For descriptive purposes, bowel preparation quality was further categorized as excellent (BBPS 8–9), good (BBPS 6–7 with all segmental scores ≥2), fair (BBPS 3–5 or BBPS 6–7 with at least one segmental score <2), or poor (BBPS 0–2). For model development and subsequent analyses, excellent and good preparation were classified as optimal, whereas fair and poor preparation were classified as suboptimal.

### 2.4 Statistical analysis

Statistical analyses were performed using SPSS Statistics version 17.0 (SPSS Inc., Chicago, IL, USA) and R software (version 4.5.3; R Foundation for Statistical Computing, Vienna, Austria). Continuous variables were expressed as mean ± standard deviation (SD) or median [interquartile range (IQR)], as appropriate, and compared using Student's t-test or the Mann–Whitney U test. Categorical variables were presented as numbers and percentages and compared using the chi-square test or Fisher's exact test, as appropriate.

Variables were assigned to two models according to their availability relative to selection of the bowel-preparation regimen. The baseline model included only patient characteristics available beforehand: age, sex, inpatient status, previous colonoscopy, family history of gastrointestinal malignancy, hypertension, diabetes mellitus, and constipation. Colonoscopy indication was available before selection of the bowel-preparation regimen but was not included in the prespecified baseline predictor set. Because the indication categories were reconstructed retrospectively from recorded examination purposes, their association with bowel-preparation quality was evaluated in a separate adjusted model rather than incorporated *post hoc* into the prediction model. The extended model added mosapride use, bowel-preparation timing, preparation agent, and linaclotide use and therefore estimated residual risk after the preparation regimen had been selected. Variables were retained according to these definitions; no univariable *P* value screening threshold was used for model inclusion. Associations are reported as odds ratios (ORs) with 95% confidence intervals (CIs). Colonoscopy indication was evaluated in a separate adjusted logistic regression model that included age, sex, inpatient status, hypertension, diabetes mellitus, previous colonoscopy, and constipation. Its overall association was tested using a likelihood-ratio test.

The primary cohort comprised all consecutively eligible patients during the study period; therefore, no *a priori* sample-size calculation was performed. There were 426 outcome events and 12 predictor parameters in the extended model, corresponding to 35.5 events per predictor parameter. The linearity of age on the logit scale was assessed by comparing the linear specification with a restricted cubic spline using a likelihood-ratio test, with a Box–Tidwell age-by-log(age) term examined as a supplementary diagnostic. Multicollinearity was assessed using variance inflation factors (VIFs), with VIF <5 considered to indicate the absence of problematic multicollinearity.

Separate multivariable logistic regression models were fitted for inadequate cleansing of the left, transverse, and right colon to describe within-segment associations. Formal differences across segments were assessed using segment-by-predictor interaction terms in long-format logistic models with patient-cluster robust covariance estimates. These analyses were exploratory. Benjamini–Hochberg-adjusted *P* values were reported for the eight interaction tests, whereas the within-segment *P* values were not adjusted for multiple testing.

The baseline and extended models were compared in terms of discrimination, calibration, overall accuracy, and net benefit. Discrimination was assessed using the area under the receiver operating characteristic curve (AUC). Internal validation used 1,000 bootstrap resamples to estimate optimism-corrected AUC, calibration intercept, calibration slope, and Brier score (18, 19). The extended model was additionally presented as a nomogram and evaluated using a calibration plot and decision curve analysis. Because it includes treatment and preparation variables, the extended model was interpreted as a residual-risk model after regimen selection rather than a model of intrinsic pretreatment risk.

Decision curve analysis (DCA) compared model-guided intensified preparation support with treat-all and treat-none strategies across a range of threshold probabilities (20). No single intensification protocol was prespecified or evaluated. Patients were also grouped as low risk (<10%), intermediate risk (10%–25%), or high risk (≥25%) using pragmatic thresholds selected for descriptive presentation. These cut-offs were not optimized from the data or validated as clinical decision thresholds. The observed proportion of suboptimal bowel preparation was compared across the three groups.

As a secondary exploratory analysis, adenoma detection was compared across model-predicted risk groups using the low-risk group as the reference. Logistic regression was used to estimate ORs for adenoma detection in the intermediate- and high-risk groups. The association between bowel preparation quality and adenoma detection was additionally evaluated using logistic regression adjusted for age and sex. Because adenoma detection was not the primary study outcome, these analyses were considered exploratory and were not incorporated into model development or internal validation.

The older comparison cohort was analysed separately as an exploratory supplementary analysis. The baseline and extended predictor sets were retained without univariable screening, and an augmented model additionally included anaesthesia and colonoscopy indication. AUC and Brier score were estimated with 1,000 bootstrap resamples. The younger-cohort extended model was also applied without refitting to the older cohort to examine transportability. Because the cohorts had different analysis exclusions, this assessment was not treated as external validation or a direct age-group comparison.

Age-related heterogeneity was examined in a separate exploratory pooled analysis of the younger and older cohorts. For each predictor available in both datasets, an age-group-by-predictor term was added separately to a logistic model containing age, age group, sex, inpatient status, previous colonoscopy, family history of gastrointestinal malignancy, hypertension, diabetes mellitus, constipation, mosapride, preparation timing, preparation agent, linaclotide, anaesthesia, and colonoscopy indication. Interaction terms were assessed using likelihood-ratio tests, with Benjamini–Hochberg adjustment across 13 tests. Because the cohorts had different analysis exclusions, these analyses were used to compare associations rather than to establish the necessity of an age-specific model.

Calendar-time effects were examined in the primary cohort using examination dates. Calendar time was grouped as 2017–2020, 2021–2023, and 2024–2025 for descriptive and sensitivity analyses. Changes in suboptimal preparation, split-dose preparation, preparation agent, linaclotide, and mosapride were summarized by period. Calendar period was then added to the baseline and extended models, and its overall contribution was assessed using likelihood-ratio tests. For internal temporal validation, models were fitted to examinations performed in 2017–2022 and applied without refitting to examinations performed in 2023–2025; discrimination, Brier score, calibration intercept, and calibration slope were evaluated in the later period.

To examine the effect of excluding severe manifestations of inadequate preparation, an outcome-related sensitivity analysis added the 42 otherwise eligible younger examinations that were incomplete because of poor preparation and the 90 otherwise eligible younger examinations requiring rescue cleansing, as described in Section 2.1. All 132 examinations were classified as having the composite adverse preparation outcome, irrespective of the post-rescue BBPS score. The baseline and extended models were refitted using their original predictor sets. Apparent and 1,000-resample optimism-corrected performance was estimated, and the originally fitted models were also applied without refitting to the expanded cohort to assess discrimination and calibration for the broader composite outcome.

All statistical tests were two-sided, and *P* values <0.05 were considered statistically significant.

### 2.5 Reporting guideline

This study was reported in accordance with the Transparent Reporting of a Multivariable Prediction Model for Individual Prognosis or Diagnosis (TRIPOD) statement for prediction model development and internal validation (21).

## 3 Results

### 3.1 Study population and baseline characteristics

A total of 2,283 individuals aged ≤50 years who underwent complete colonoscopy were included in the final analysis. Of these, 1,857 (81.3%) had optimal bowel preparation and 426 (18.7%) had suboptimal bowel preparation according to the predefined BBPS criteria.

Baseline characteristics are summarized in Table 1. Colonoscopy indications were polyp surveillance in 189 participants (8.3%), family history in 329 (14.4%), symptomatic investigation in 859 (37.6%), and screening in 906 (39.7%). The corresponding suboptimal-preparation rates were 18.5%, 18.8%, 17.5%, and 19.8%, respectively. After adjustment for age, sex, inpatient status, hypertension, diabetes mellitus, previous colonoscopy, and constipation, colonoscopy indication was not associated with suboptimal bowel preparation as an overall factor (likelihood-ratio *P* = 0.358). Diabetes mellitus and constipation were more frequent, whereas split-dose preparation and linaclotide use were less frequent, among participants with suboptimal preparation.

**Table 1:** Baseline characteristics of the study population stratified by bowel-preparation quality.

Variables	All patients (*n* = 2,283)	Optimal (*n* = 1,857)	Suboptimal (*n* = 426)	*P*-value
Patient characteristics				
Age (years)	40.11 ± 7.36	39.91 ± 7.45	40.98 ± 6.90	0.007
Males, *n* (%)	1,156 (50.64)	934 (50.30)	222 (52.11)	0.499
Patient setting, *n* (%)				
Inpatient	627 (27.46)	519 (27.95)	108 (25.35)	0.279
Outpatient	1,656 (72.54)	1,338 (72.05)	318 (74.65)	
Colonoscopy history, *n* (%)	480 (21.02)	391 (21.06)	89 (20.89)	0.940
Family history of gastrointestinal malignancy, *n* (%)	272 (11.91)	217 (11.69)	55 (12.91)	0.481
Colonoscopy indication				0.673
Polyp surveillance, *n* (%)	189 (8.28)	154 (8.29)	35 (8.22)	
Family history, *n* (%)	329 (14.41)	267 (14.38)	62 (14.55)	
Symptomatic investigation, *n* (%)	859 (37.63)	709 (38.18)	150 (35.21)	
Screening, *n* (%)	906 (39.68)	727 (39.15)	179 (42.02)	
Comorbidities				
Hypertension, *n* (%)	106 (4.64)	83 (4.47)	23 (5.40)	0.411
Diabetes, *n* (%)	196 (8.59)	80 (4.31)	116 (27.23)	<0.001
Constipation, *n* (%)	262 (11.47)	127 (6.84)	135 (31.69)	<0.001
*Preparation-related factors*				
Bowel preparation method				
Same-day preparation, *n* (%)	985 (43.14)	730 (39.31)	255 (59.86)	<0.001
Split-dose preparation, *n* (%)	1,298 (56.86)	1,127 (60.69)	171 (40.14)	
Bowel preparation agents				
PEG-electrolyte solution, *n* (%)	1,477 (64.70)	1,189 (64.03)	288 (67.61)	0.163
Mannitol, *n* (%)	806 (35.30)	668 (35.97)	138 (32.39)	
Anesthesia use, *n* (%)	2,078 (91.02)	1,695 (91.28)	383 (89.91)	0.397
Mosapride use, *n* (%)	120 (5.26)	95 (5.12)	25 (5.87)	0.530
Linaclotide use, *n* (%)	138 (6.04)	125 (6.73)	13 (3.05)	0.004

Data are presented as mean ± standard deviation for continuous variables and number (%) for categorical variables. Unadjusted *P* values were calculated using Student's t-test, the chi-square test, or Fisher's exact test, as appropriate. For variables with more than two categories, including colonoscopy indication, the reported *P* value represents the global comparison across all categories. Colonoscopy indication was also evaluated in a separate multivariable logistic regression model; its adjusted overall association was tested using a likelihood-ratio test (*P* = 0.358). In the source database, anaesthesia was coded as 0 for anaesthesia and 1 for no anaesthesia; the counts shown here were corrected so that “anaesthesia use” denotes receipt of anaesthesia. Suboptimal bowel preparation was defined as a total BBPS score <6 or any segmental score <2.

BBPS, Boston Bowel Preparation Scale; PEG, polyethylene glycol-electrolyte solution; GI, gastrointestinal.

No statistically significant between-group differences were observed in sex, patient setting, previous colonoscopy, family history of gastrointestinal malignancy, hypertension, bowel-preparation agent, anaesthesia or sedation use, mosapride use, or colonoscopy indication.

### 3.2 Identification of predictors for suboptimal bowel preparation

The results of the univariable and multivariable logistic regression analyses are presented in Table 2, and the adjusted effect estimates from the full multivariable model are shown in Figure 2. In univariable analyses, older age, diabetes mellitus, constipation, same-day bowel preparation, and non-use of linaclotide were associated with suboptimal bowel preparation (all *P* < 0.05). Sex, patient setting, previous colonoscopy, family history of gastrointestinal malignancy, hypertension, bowel preparation agent, anesthesia or sedation use, and mosapride use were not significantly associated with bowel preparation quality.

**Table 2:** Univariable and multivariable logistic regression analyses for predictors of suboptimal bowel preparation.

Candidate predictors	Univariate analysis		Multivariable analysis	
	OR (95% CI)	*P*-value	OR (95% CI)	*P*-value
Age (per year)	1.021 (1.006- 1.036)	0.007	1.002 (0.986–1.020)	0.688
Male sex (vs. female)	1.075 (0.871–1.328)	0.499	0.772 (0.605–0.986)	0.038
Patient setting (reference: Outpatient)				
Inpatient	0.876 (0.688- 1.114)	0.279	0.529 (0.388–0.721)	<0.001
Previous colonoscopy	0.990 (0.764–1.283)	0.940	1.257 (0.936–1.688)	0.129
Family history of gastrointestinal malignancy	1.114 (0.810–1.533)	0.506	1.090 (0.764–1.555)	0.636
Hypertension	1.220 (0.759–1.960)	0.412	0.711 (0.399–1.265)	0.246
Diabetes	8.312 (6.100- 11.325)	<0.001	10.701 (7.312–15.660)	<0.001
Constipation	6.320 (4.813- 8.297)	<0.001	6.250 (4.619–8.458)	<0.001
Mosapride use	1.156 (0.735- 1.820)	0.530	1.452 (0.869–2.425)	0.155
Anesthesia or sedation use	0.851 (0.597–1.213)	0.373	–	–
Linaclotide use	0.436 (0.244- 0.780)	0.005	0.375 (0.196–0.718)	0.003
Split-dose vs. Same-day	0.434 (0.351–0.537)	<0.001	0.459 (0.362–0.583)	<0.001
PEG vs. mannitol	1.172 (0.937- 1.467)	0.164	1.017 (0.773–1.339)	0.902

Univariable logistic regression analyses are shown descriptively. The extended multivariable model used the prespecified predictor set shown in the table and was not selected using a univariable *P* value threshold. The baseline model excluded the four preparation and treatment variables and is compared with the extended model in Table 3. Results are presented as ORs with 95% CIs.

OR, odds ratio; CI, confidence interval; PEG, polyethylene glycol-electrolyte solution.

All statistical tests were two-sided, and *P* values <0.05 were considered statistically significant.

**Figure 2:**
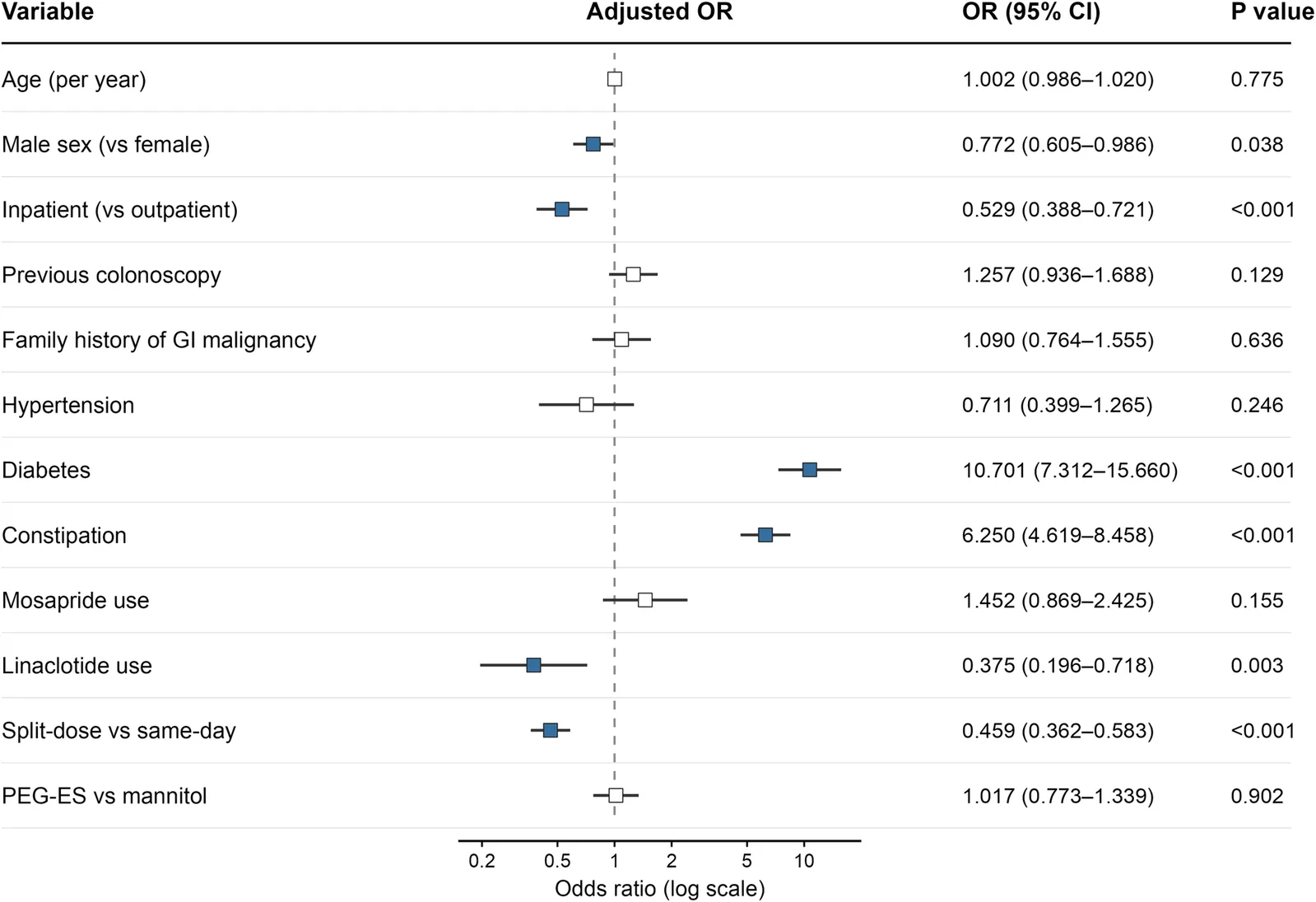
Forest plot of the final extended multivariable logistic regression model for suboptimal bowel preparation. Adjusted ORs with 95% CIs are shown on a logarithmic scale. Filled squares denote *P* < 0.05; open squares denote *P* ≥ 0.05. The dashed line represents OR = 1.

In the extended model, diabetes mellitus (adjusted OR: 10.701, 95% CI: 7.312–15.660; *P* < 0.001) and constipation (adjusted OR: 6.250, 95% CI: 4.619–8.458; *P* < 0.001) showed the strongest positive associations with suboptimal bowel preparation. Split-dose preparation was associated with lower odds than same-day preparation (adjusted OR: 0.459, 95% CI: 0.362–0.583; *P* < 0.001). Linaclotide use was also associated with lower odds (adjusted OR: 0.375, 95% CI: 0.196–0.718; *P* = 0.003).

Male sex (adjusted OR: 0.772, 95% CI: 0.605–0.986; *P* = 0.038) and inpatient status (adjusted OR: 0.529, 95% CI: 0.388–0.721; *P* < 0.001) were associated with lower odds of suboptimal bowel preparation. Age, previous colonoscopy, family history of gastrointestinal malignancy, hypertension, preparation agent, and mosapride use were not independently associated with the outcome.

The extended model was used for the nomogram and subsequent residual-risk analyses.

### 3.3 Development and performance of the prediction model

The baseline model contained only patient characteristics available before selection of the bowel-preparation regimen. The extended model added mosapride, preparation timing, preparation agent, and linaclotide and was identical to the original full multivariable model used for the nomogram and risk-stratification analyses.

The apparent AUC was 0.722 for the baseline model and 0.761 for the extended model (Figure 3 and Table 3). The addition of preparation and treatment variables increased the AUC by 0.039 and improved likelihood fit (likelihood-ratio *P* < 0.001).

**Figure 3:**
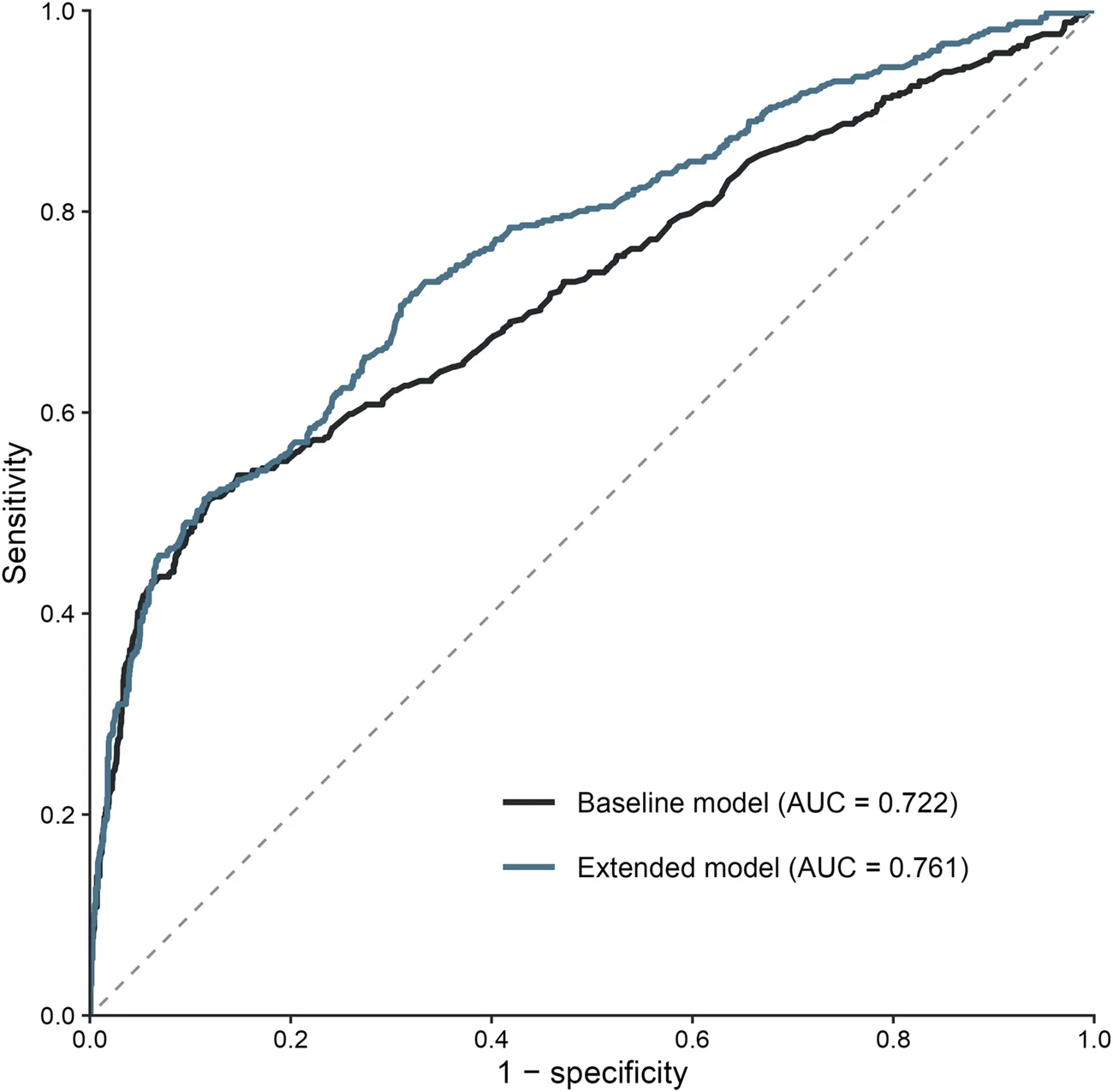
Receiver operating characteristic curves for the baseline and extended prediction models. The baseline model contains patient characteristics available before bowel-preparation regimen selection. The extended model additionally contains preparation and treatment variables and estimates residual risk after regimen selection. AUC, area under the receiver operating characteristic curve.

**Table 3:** Predictive performance and internal validation of the baseline and extended prediction models.

Domain	Metric	Baseline model	Extended model
Sample size	Participants/events	2,283/426	2,283/426
Discrimination	Apparent AUC	0.722	0.761
Bootstrap validation	Optimism-corrected AUC	0.714	0.752
Overall accuracy	Apparent Brier score	0.123	0.120
Bootstrap validation	Optimism-corrected Brier score	0.125	0.122
Calibration	Optimism-corrected intercept	−0.030	−0.047
Calibration	Optimism-corrected slope	0.975	0.964
Model comparison	*Δ*AUC vs. baseline	Reference	0.039
Model comparison	Likelihood-ratio *P* value	Reference	<0.001

AUC, area under the receiver operating characteristic curve.

Both models were internally validated using 1,000 bootstrap resamples. The baseline model contains patient characteristics available before bowel-preparation regimen selection. The extended model adds mosapride use, preparation timing, preparation agent, and linaclotide use and estimates residual risk after regimen selection. Lower Brier scores indicate better overall accuracy; ideal calibration intercept and slope values are 0 and 1, respectively.

After 1,000 bootstrap resamples, the optimism-corrected AUC was 0.714 for the baseline model and 0.752 for the extended model. The corresponding optimism-corrected Brier scores were 0.125 and 0.122, calibration intercepts were −0.030 and −0.047, and calibration slopes were 0.975 and 0.964. Thus, adding preparation-related variables produced a modest improvement in discrimination and overall accuracy while maintaining similar calibration (Table 3).

The baseline model can be applied before the bowel-preparation regimen is selected. The extended model can be applied after regimen selection to estimate residual risk. The complete model specifications, including intercepts, six-decimal logistic regression coefficients, predictor coding, and equations for calculating individual predicted probabilities, are provided in Supplementary Table S1.

There was no evidence that the linear age specification was inadequate (restricted-cubic-spline likelihood-ratio *P* = 0.308; Box–Tidwell *P* = 0.239). VIFs ranged from 1.008 to 1.109 in the baseline model and from 1.013 to 1.281 in the extended model, indicating no problematic multicollinearity (Supplementary Table S2).

### 3.4 Nomogram construction, calibration, and exploratory decision analysis

A nomogram was constructed from the extended multivariable logistic regression model to estimate residual risk after the bowel-preparation regimen had been selected (Figure 4). Each predictor was assigned a point value according to its regression coefficient, and total points corresponded to the predicted probability of suboptimal bowel preparation.

**Figure 4:**
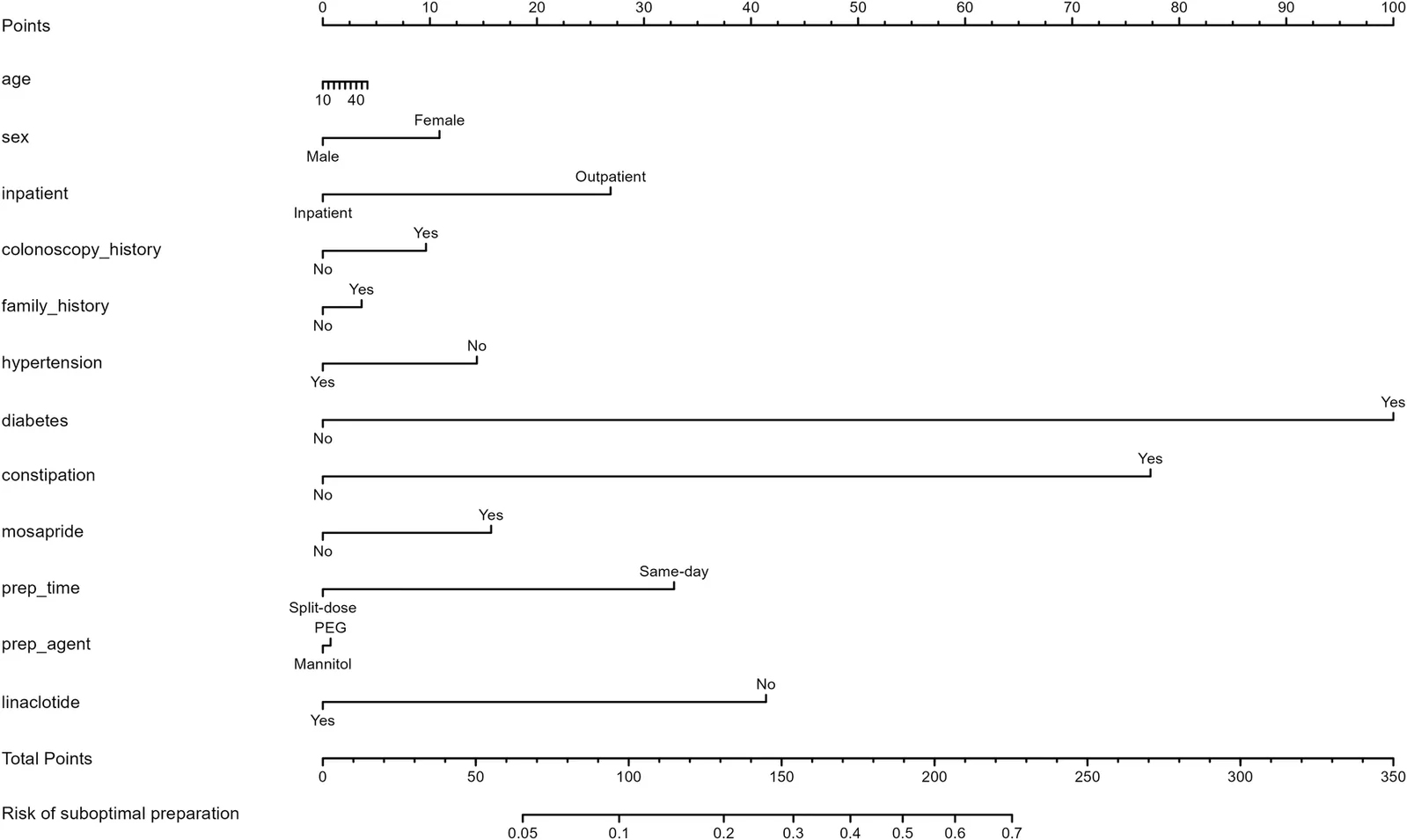
Nomogram for individualized residual-risk prediction based on the extended multivariable logistic regression model after selection of the bowel-preparation regimen.

The bootstrap-corrected calibration curve generally tracked the ideal line, with greater variability at lower predicted risks (Figure 5).

**Figure 5:**
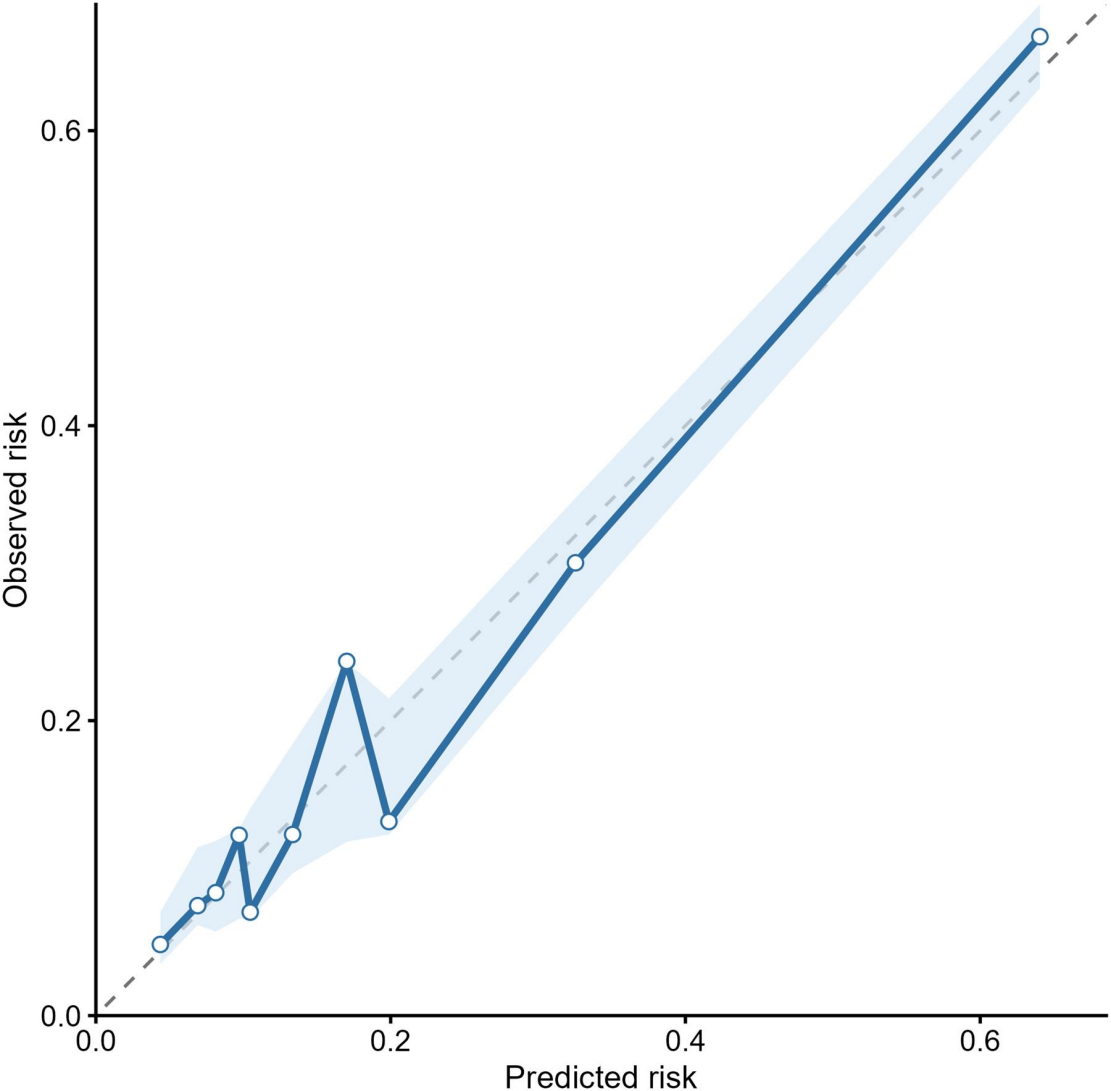
Bootstrap-corrected calibration plot of the extended prediction model. The dashed line represents perfect calibration.

Decision curve analysis compared the extended model with treat-all and treat-none strategies (Figure 6). Within the plotted low-to-moderate threshold range, the model showed greater net benefit than either default strategy. A threshold probability denotes the minimum predicted risk at which intensified preparation support would be considered. Because no single intensification protocol was prespecified or tested, this analysis remains exploratory and does not establish clinical utility.

**Figure 6:**
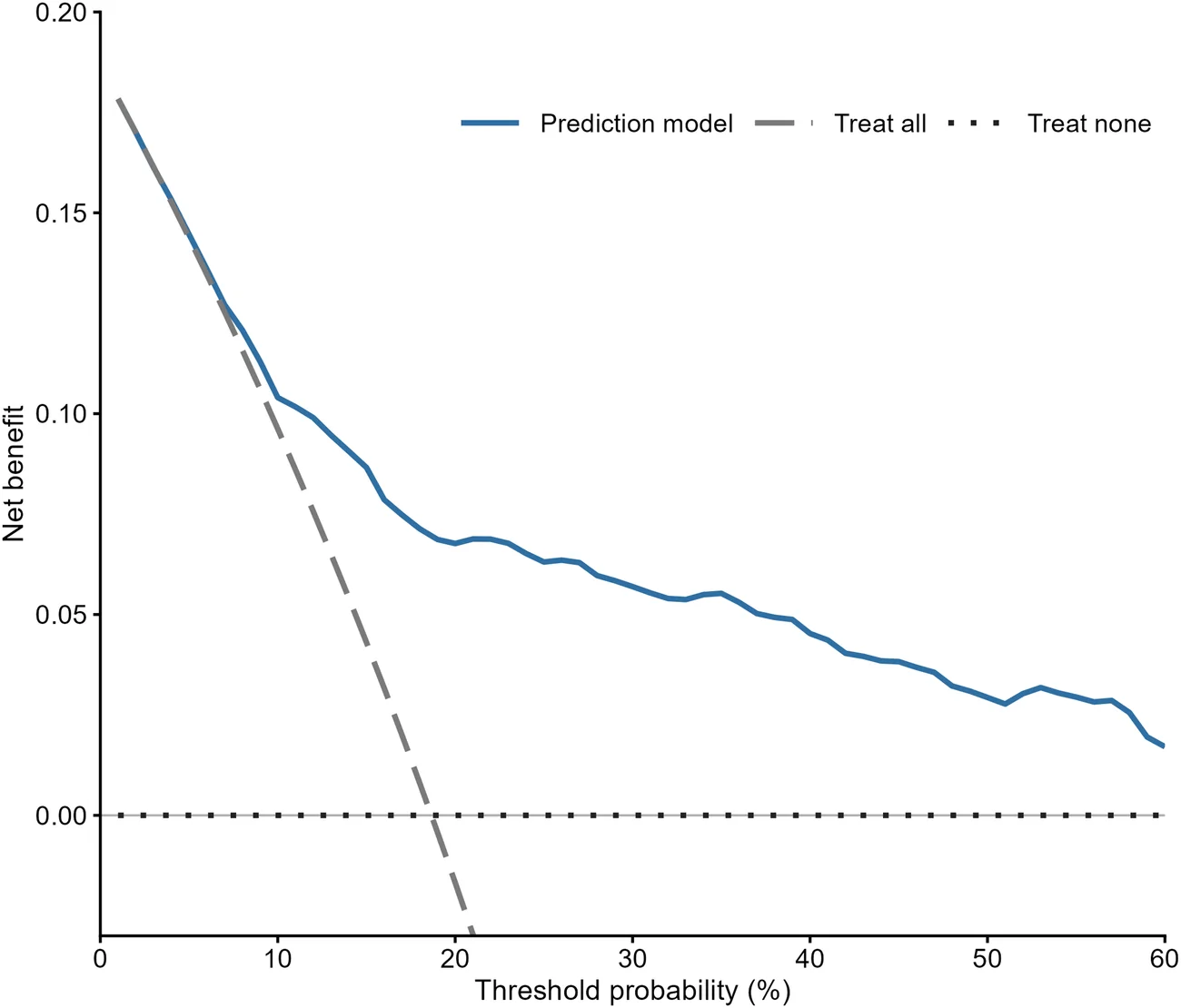
Decision curve analysis of the extended prediction model. Net benefit is shown across threshold probabilities and compared with treat-all and treat-none strategies. Threshold probability denotes the minimum predicted risk at which intensified preparation support would be considered.

Patients were further stratified using probabilities from the extended model into low-risk (<10%), intermediate-risk (10%–25%), and high-risk (≥25%) groups. As shown in Figure 7, the observed rate of suboptimal bowel preparation increased across the three groups: 8.2%, 14.7%, and 51.7%, respectively.

**Figure 7:**
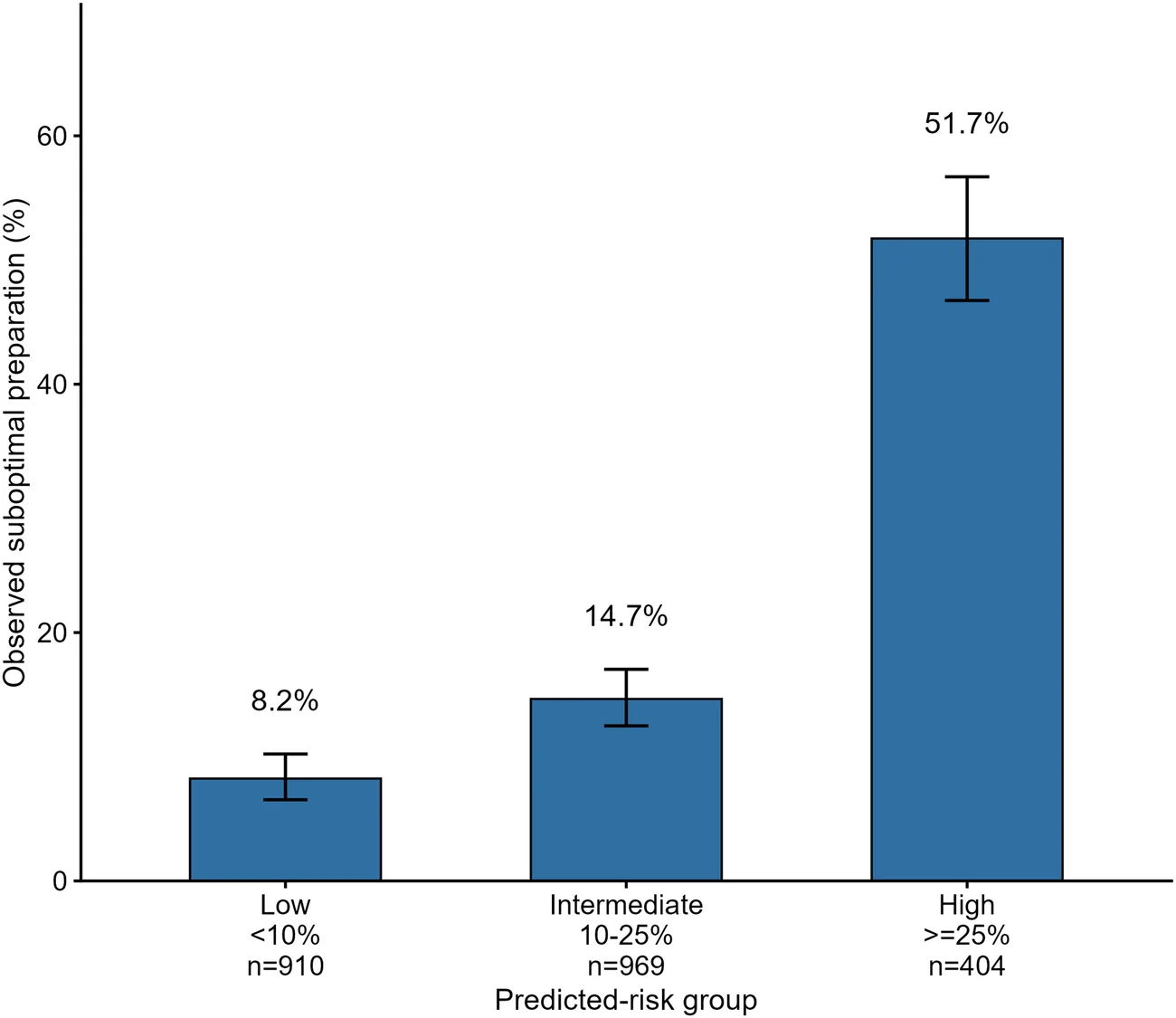
Observed rate of suboptimal bowel preparation across predefined risk groups derived from the extended model. Error bars indicate 95% confidence intervals. The thresholds are exploratory and require external validation.

The extended model therefore provided descriptive residual-risk stratification after regimen selection. These thresholds were not selected by data-driven optimization and cannot be used as clinical decision thresholds without prospective validation and a prespecified clinical intervention.

### 3.5 Segment-specific analyses of predictors for suboptimal bowel preparation

Segment-specific multivariable logistic regression results are presented in Table 4. Formal segment-by-predictor interaction tests showed evidence that the association with diabetes mellitus differed across colonic segments (Wald χ^2^ = 44.137, df = 2; unadjusted and Benjamini–Hochberg-adjusted *P* < 0.001; Supplementary Table S3). No other interaction remained significant after adjustment for the eight interaction tests (adjusted *P* = 0.066–0.762).

**Table 4:** Segment-specific multivariable logistic regression analyses for predictors of suboptimal bowel preparation.

Variables	Left colon OR (95% CI)	*P*-value	Transverse colon OR (95% CI)	*P*-value	Right colon OR (95% CI)	*P*-value
Male sex (vs. female)	0.722 (0.437–1.194)	0.204	1.251 (0.827–1.891)	0.289	0.846 (0.658–1.088)	0.193
Age (per year)	0.981 (0.949–1.015)	0.277	1.019 (0.990–1.050)	0.204	1.005 (0.987–1.023)	0.599
Previous colonoscopy	1.215 (0.652–2.263)	0.540	1.441 (0.898–2.312)	0.130	1.186 (0.874–1.609)	0.272
Diabetes mellitus	1.917 (0.965–3.810)	0.063	0.825 (0.400–1.703)	0.603	8.208 (5.768–11.678)	<0.001
Constipation	6.851 (4.128–11.369)	<0.001	4.835 (3.102–7.536)	<0.001	3.865 (2.842–5.256)	<0.001
Split-dose preparation (vs. same-day)	0.610 (0.368–1.000)	0.052	0.636 (0.421–0.956)	0.030	0.455 (0.354–0.582)	<0.001
PEG-ES (vs. mannitol)	1.287 (0.736–2.251)	0.377	0.661 (0.433–1.010)	0.055	0.796 (0.608–1.042)	0.097
Linaclotide use	0.171 (0.023–1.271)	0.084	0.281 (0.067–1.178)	0.083	0.444 (0.226–0.874)	0.019

Multivariable logistic regression analyses were performed separately for the left, transverse, and right colon. Each model simultaneously included sex, age, previous colonoscopy, diabetes mellitus, constipation, preparation timing, preparation agent, and linaclotide use. Within-segment results are exploratory and their *P* values were not adjusted for multiple testing. Statistical significance in one segment but not another was not interpreted as evidence of a difference between segments. Formal segment-by-predictor comparisons, with Benjamini–Hochberg correction across eight interaction tests, are reported in Supplementary Table S3.

OR, odds ratio; CI, confidence interval; PEG-ES, polyethylene glycol-electrolyte solution.

A two-sided *P* value <0.05 was considered statistically significant.

In the exploratory within-segment models, constipation was associated with inadequate cleansing in the left colon (OR: 6.851, 95% CI: 4.128–11.369; *P* < 0.001), transverse colon (OR: 4.835, 95% CI: 3.102–7.536; *P* < 0.001), and right colon (OR: 3.865, 95% CI: 2.842–5.256; *P* < 0.001). The constipation-by-segment interaction was not significant (adjusted *P* = 0.421).

Diabetes mellitus was associated with inadequate cleansing in the right colon (OR: 8.208, 95% CI: 5.768–11.678; *P* < 0.001), but not in the left or transverse colon; this pattern was supported by the formal interaction test reported above.

Compared with same-day preparation, split-dose preparation was associated with lower odds of inadequate cleansing in the transverse colon (OR: 0.636, 95% CI: 0.421–0.956; *P* = 0.030) and right colon (OR: 0.455, 95% CI: 0.354–0.582; *P* < 0.001), whereas the within-segment estimate for the left colon was OR: 0.610 (95% CI: 0.368–1.000; *P* = 0.052). The preparation-timing-by-segment interaction was not significant (adjusted *P* = 0.421), so these within-segment *P* values were not interpreted as evidence of different effects across segments.

### 3.6 Exploratory analysis of adenoma detection according to prediction model risk groups

The association between prediction model risk groups and adenoma detection was explored as a secondary analysis (Supplementary Table S1). Patients were categorized into low-risk (<10%), intermediate-risk (10%–25%), and high-risk (≥25%) groups according to the predicted probability of suboptimal bowel preparation generated by the final model.

The observed adenoma detection rates were 20.9% (190/910), 16.2% (157/969), and 19.6% (79/404) in the low-, intermediate-, and high-risk groups, respectively. In unadjusted logistic regression using the low-risk group as the reference, the intermediate-risk group had lower odds of adenoma detection (OR: 0.733, 95% CI: 0.580–0.926; *P* = 0.009), whereas the high-risk group did not differ significantly from the low-risk group (OR: 0.921, 95% CI: 0.687–1.235; *P* = 0.583).

In a separate exploratory analysis adjusted for age and sex, suboptimal bowel preparation was associated with lower odds of adenoma detection compared with optimal bowel preparation (OR: 0.712, 95% CI: 0.532–0.953; *P* = 0.023).

Because adenoma detection was not the primary outcome of this study, these findings should be interpreted cautiously. The model-predicted risk groups were not associated with a monotonic increase or decrease in adenoma detection, indicating that the model should be interpreted as a tool for predicting suboptimal bowel preparation rather than adenoma risk. The association between suboptimal bowel preparation and lower adenoma detection supports the clinical importance of adequate bowel cleansing.

### 3.7 Exploratory analysis in the older comparison cohort

Among 3,146 participants aged >50 years, 653 (20.8%) had suboptimal bowel preparation (Supplementary Table S5). In the extended model, diabetes mellitus (OR: 3.692, 95% CI: 2.924–4.662), constipation (OR: 4.326, 95% CI: 3.486–5.368), and mosapride use (OR: 4.594, 95% CI: 3.262–6.470) were associated with higher odds of suboptimal preparation (all *P* < 0.001). Split-dose preparation was associated with lower odds (OR: 0.618, 95% CI: 0.511–0.748; *P* < 0.001).

Colonoscopy indication was not associated with the outcome after adjustment (global likelihood-ratio *P* = 0.844). The extended model had an apparent AUC of 0.748 and an optimism-corrected AUC of 0.742. Adding anaesthesia and colonoscopy indication did not improve model fit (likelihood-ratio *P* = 0.887). When the younger-cohort extended model was applied without refitting, the AUC was 0.729 and the calibration slope was 0.663.

### 3.8 Exploratory age-group comparison and interaction analysis

Suboptimal bowel preparation occurred in 426 of 2,283 participants aged ≤50 years (18.7%) and 653 of 3,146 participants aged >50 years (20.8%; unadjusted *P* = 0.056). In common-covariate models, formal age-group interactions were observed for diabetes mellitus, mosapride, and linaclotide (Benjamini–Hochberg-adjusted *P* < 0.001, <0.001, and 0.045, respectively; Supplementary Table S6). The adjusted association with diabetes was stronger in the younger cohort (OR: 10.906, 95% CI: 7.441–15.985) than in the older cohort (OR 3.712, 95% CI: 2.939–4.689). Mosapride was not associated with the outcome in the younger cohort (OR: 1.459, 95% CI: 0.873–2.439) but was associated with higher odds in the older cohort (OR: 4.554, 95% CI: 3.231–6.418). Linaclotide was associated with lower odds in the younger cohort (OR: 0.372, 95% CI: 0.194–0.713) but not in the older cohort (OR: 0.929, 95% CI: 0.656–1.315). No evidence of age-group interaction remained for the other assessed predictors after multiplicity adjustment.

### 3.9 Calendar-time sensitivity analysis and internal temporal validation

The suboptimal-preparation rate was 10.3% in 2017–2020, 16.7% in 2021–2023, and 41.2% in 2024–2025. Over the same periods, split-dose use decreased from 61.8% to 56.1% and 48.2%, while PEG-ES use was 58.8%, 55.3%, and 100%, respectively. Linaclotide use was 3.1%, 9.3%, and 4.6%, and mosapride use was 5.6%, 4.1%, and 7.3% (Supplementary Table S7). Calendar period improved both the baseline and extended models (likelihood-ratio *P* < 0.001 for each). In the calendar-adjusted extended model, diabetes mellitus (OR: 6.174, 95% CI: 4.109–9.277), constipation (OR: 5.163, 95% CI: 3.781–7.050), split-dose preparation (OR: 0.483, 95% CI: 0.379–0.617), and linaclotide use (OR: 0.372, 95% CI: 0.194–0.716) retained their directions and statistical significance.

For internal temporal validation, the 2017–2022 development sample included 1,559 participants and 224 events, and the 2023–2025 validation sample included 724 participants and 202 events. In the later period, the baseline and extended models had AUCs of 0.767 and 0.769 and Brier scores of 0.155 and 0.157, respectively. The calibration intercepts were 0.368 and 0.389, and the calibration slopes were 0.888 and 0.830, indicating systematic underestimation of risk in the later period (Supplementary Table S7). This chronological assessment was internal and was not considered external validation.

### 3.10 Outcome-related sensitivity analysis

After the remaining eligibility criteria were applied, 42 younger examinations that were incomplete because of poor preparation and 90 that required rescue cleansing were added to the 2,283-examination primary cohort. The expanded cohort therefore included 2,415 examinations and 558 composite outcome events (23.1%; Supplementary Table S8). In the refitted extended model, diabetes mellitus (OR: 8.039, 95% CI: 5.768–11.205), constipation (OR: 5.651, 95% CI: 4.289–7.445), and split-dose preparation (OR: 0.503, 95% CI: 0.407–0.622) retained their directions and statistical significance. Linaclotide remained inversely associated, although its confidence interval included the null (OR 0.638, 95% CI 0.392–1.040; *P* = 0.071).

The refitted baseline and extended models had apparent AUCs of 0.704 and 0.747 and optimism-corrected AUCs of 0.691 and 0.740, respectively. When the original models were applied without refitting to the expanded cohort, the baseline and extended AUCs were 0.683 and 0.726. For the original extended model, the calibration intercept was 0.125 and the calibration slope was 0.880, indicating underestimation of the broader composite risk.

## 4 Discussion

Colonoscopy remains central to colorectal cancer prevention and early detection, and its diagnostic yield depends on bowel-preparation quality. In this retrospective cohort of individuals aged ≤50 years, suboptimal preparation occurred in 18.7% of examinations. Our outcome definition required a total BBPS score <6 or any segmental score <2; thus, an examination with an acceptable total score but inadequate cleansing in one segment was classified as suboptimal. The tertiary-care case mix may also have contributed, because 27.5% of participants were inpatients and 8.6% had diabetes mellitus. Under the local clinical protocol, some afternoon examinations used same-day preparation. However, procedure times and individual reasons for regimen selection were not recorded prospectively, so their contribution to the observed rate cannot be quantified. Diabetes-related autonomic dysfunction and impaired gastrointestinal motility provide a plausible explanation for difficult cleansing, but this mechanism was not measured here. Constipation and diabetes mellitus were associated with higher odds, whereas split-dose preparation and linaclotide use were associated with lower odds.

This study separated two clinically distinct prediction times. The baseline model used patient characteristics available before regimen selection and showed an optimism-corrected AUC of 0.714. The extended model added preparation and treatment variables and showed a corrected AUC of 0.752. The modest increment in discrimination indicates that the selected regimen contains additional prognostic information. However, this extended model estimates residual risk after treatment selection and should not be described as predicting intrinsic pretreatment risk. Its apparent net benefit and risk stratification require external validation before implementation.

Constipation was associated with suboptimal bowel preparation and with inadequate cleansing in all three colonic segments. The constipation-by-segment interaction was not significant, so the numerical differences between segment-specific odds ratios do not provide evidence of regional heterogeneity. This finding is consistent with previous reports linking constipation to inadequate preparation (9, 12, 22). Delayed colonic transit and stool retention may reduce the effectiveness of bowel-cleansing agents (22, 23). Reinforced instructions and individualized support may be reasonable for patients with constipation, but this approach requires prospective evaluation.

Diabetes mellitus was independently associated with suboptimal bowel preparation in the overall model. Formal interaction analysis also supported variation across colonic segments, with the clearest association observed for right-colon cleansing. This finding is consistent with previous reports linking diabetes to poorer bowel-preparation quality (9, 24, 25). Potential explanations include autonomic neuropathy, delayed gastrointestinal transit, and impaired colonic motility (26, 27). although these mechanisms were not measured in the present study. The segment-specific finding remains exploratory and requires prospective confirmation.

Linaclotide is a guanylate cyclase-C agonist that increases intestinal fluid secretion and accelerates bowel transit, providing a plausible mechanism for improved bowel cleansing (13, 28). In the present study, Linaclotide use was associated with lower odds of suboptimal bowel preparation in the overall model. The formal linaclotide-by-segment interaction was not significant; therefore, the separate segment-specific estimates were not interpreted as evidence that its association differed across the colon. Linaclotide use was not randomized, and residual confounding by indication or patient selection cannot be excluded. These estimates remain hypothesis-generating, and prospective studies are needed before any treatment effect is inferred.

Clinically, the baseline model could support risk assessment before the bowel-preparation regimen is selected. The extended model could then estimate residual risk after regimen selection and identify patients who may still require additional support. Possible strategies include reinforced education, closer assessment of bowel habits, and optimization of preparation timing. Prospective studies should evaluate whether this two-stage approach improves bowel cleanliness or reduces repeat examinations.

The older-cohort analysis showed similar directions for diabetes mellitus, constipation, and split-dose preparation. Mosapride was associated with higher odds in the older cohort, whereas linaclotide was not associated with preparation quality. When applied to the older cohort, the younger-cohort model retained moderate discrimination but had a calibration slope of 0.663. Formal interaction tests indicated age-related heterogeneity for diabetes, mosapride, and linaclotide, but not for the other predictors. These comparisons are exploratory because the older cohort had additional exclusions and lacked examination dates and segmental BBPS scores. The model is therefore best described as developed in a younger colonoscopy population, rather than as superior to a general-adult model.

Preparation practice and bowel preparation quality varied substantially over calendar time. The suboptimal-preparation rate increased from 10.3% in 2017–2020 to 41.2% in 2024–2025, accompanied by a decline in split-dose preparation and a shift away from mannitol toward PEG-ES as the bowel-preparation agent. These changes coincided with local practice adjustments beginning in 2024, including a greater proportion of younger patients being scheduled for afternoon colonoscopy, which may have increased the use of same-day preparation. During the same period, the coexistence of gastroenterology and endocrinology services on the same ward may also have altered the case mix, with more young inpatients with diabetes and metabolic comorbidities undergoing colonoscopy. A concurrent shift in the department's predominant disease profile, with greater emphasis on constipation as a major clinical condition, may also have contributed to changes in the proportion of patients with constipation in the study cohort. Diabetes-related gastrointestinal dysmotility may further compromise bowel cleansing (25), and the interaction between diabetic gastrointestinal dysfunction and preparation timing (29)warrants further investigation. However, diabetes-related gastrointestinal dysfunction, reasons for regimen selection, and the exact preparation-to-colonoscopy interval were not systematically recorded, and the independent contributions of these factors could not be determined. Calendar-period adjustment attenuated several model coefficients without changing their directions. Internal temporal validation maintained good discrimination but showed systematic underestimation of risk in the later period, suggesting temporal drift and supporting the need for model updating and external validation before clinical implementation.

The study included a relatively large age-specific cohort, standardized BBPS assessment, and complementary evaluation of discrimination, calibration, overall accuracy, and net benefit. Bootstrap validation quantified optimism, while formal interaction tests distinguished segment-specific differences from isolated within-segment significance.

The retrospective single-centre design leaves potential selection bias and limits generalisability. Data on adherence, dietary compliance, education, the exact preparation-to-colonoscopy interval, stool characteristics, body mass index, smoking, and tolerability were unavailable. Preparation regimens and adjuncts were selected by treating physicians without a written risk-stratification protocol, and the clinical reasons were not recorded prospectively. Confounding by indication and residual treatment-selection bias therefore remain possible. Interobserver variability in BBPS assessment could not be fully evaluated, and the risk thresholds require external validation. The cohort included screening, surveillance, family-history, and symptomatic examinations and should not be interpreted as an exclusively screening population. The principal model was developed among individuals aged ≤50 years who completed a standard preparation protocol without rescue cleansing and underwent complete colonoscopy; its absolute probabilities apply to that target population and to the pooled 2017–2025 case mix. The outcome-related sensitivity analysis reduced concern that exclusion of severe preparation failures reversed the principal associations, but performance was lower for the broader composite outcome and the original model underestimated its absolute risk. Substantial calendar drift provides a separate reason for recalibration and model updating before contemporary use. Examination dates were unavailable for the older cohort, precluding a same-period calendar adjustment across age groups.

In conclusion, patient characteristics available before regimen selection provided moderate discrimination for suboptimal bowel preparation in individuals aged ≤50 years. Adding preparation and treatment variables modestly improved performance and yielded an extended residual-risk model for use after regimen selection. Diabetes mellitus and constipation were associated with higher odds, whereas split-dose preparation and linaclotide use were associated with lower odds. Because regimen selection was not randomized, the treatment-related associations are not causal estimates. Independent multicentre validation and model updating are required before clinical use.

## Data Availability

The raw data supporting the conclusions of this article will be made available by the authors, without undue reservation.
