# Supplementary material for: Development and internal validation of a prediction model for suboptimal bowel preparation in individuals aged ≤50 years undergoing colonoscopy
Source: Front Med (Lausanne). 2026 Sep 23;13:1939670. doi: 10.3389/fmed.2026.1939670 (PMC13642568; doi:10.3389/fmed.2026.1939670)
Supplement: Supplementary file 1 [file Table1.docx]

Supplementary Material

# 1 Supplementary Tables

**Supplementary Table S1. Complete specification of the baseline and extended logistic prediction models**

A. **Baseline model**

| Predictor and coding | β coefficient | SE | OR | 95% CI | P value |
| --- | --- | --- | --- | --- | --- |
| Intercept | -2.057672 | 0.344684 | — | — | <0.001 |
| Age, per year | 0.004759 | 0.008401 | 1.005 | 0.988–1.021 | 0.571 |
| Male sex (vs female) | -0.212916 | 0.121991 | 0.808 | 0.636–1.027 | 0.081 |
| Inpatient setting (vs outpatient) | -0.618411 | 0.147027 | 0.539 | 0.404–0.719 | <0.001 |
| Previous colonoscopy (yes vs no) | 0.263066 | 0.147706 | 1.301 | 0.974–1.738 | 0.075 |
| Family history of gastrointestinal malignancy (yes vs no) | 0.124892 | 0.178602 | 1.133 | 0.798–1.608 | 0.484 |
| Hypertension (yes vs no) | -0.331521 | 0.291792 | 0.718 | 0.405–1.272 | 0.256 |
| Diabetes mellitus (yes vs no) | 2.359884 | 0.188019 | 10.590 | 7.326–15.308 | <0.001 |
| Constipation (yes vs no) | 1.808955 | 0.149702 | 6.104 | 4.552–8.186 | <0.001 |

B. **Extended model**

| Predictor and coding | β coefficient | SE | OR | 95% CI | P value |
| --- | --- | --- | --- | --- | --- |
| Intercept | -1.522999 | 0.359131 | — | — | <0.001 |
| Age, per year | 0.002469 | 0.008625 | 1.002 | 0.986–1.020 | 0.775 |
| Male sex (vs female) | -0.258267 | 0.124376 | 0.772 | 0.605–0.986 | 0.038 |
| Inpatient setting (vs outpatient) | -0.637191 | 0.158137 | 0.529 | 0.388–0.721 | <0.001 |
| Previous colonoscopy (yes vs no) | 0.228439 | 0.150552 | 1.257 | 0.936–1.688 | 0.129 |
| Family history of gastrointestinal malignancy (yes vs no) | 0.085842 | 0.181435 | 1.090 | 0.764–1.555 | 0.636 |
| Hypertension (yes vs no) | -0.341115 | 0.294101 | 0.711 | 0.399–1.265 | 0.246 |
| Diabetes mellitus (yes vs no) | 2.370335 | 0.194263 | 10.701 | 7.312–15.660 | <0.001 |
| Constipation (yes vs no) | 1.832622 | 0.154323 | 6.250 | 4.619–8.458 | <0.001 |
| Mosapride use (yes vs no) | 0.372605 | 0.261843 | 1.452 | 0.869–2.425 | 0.155 |
| Split-dose preparation (vs same-day) | -0.777685 | 0.121925 | 0.459 | 0.362–0.583 | <0.001 |
| PEG-ES (vs mannitol) | 0.017202 | 0.140343 | 1.017 | 0.773–1.339 | 0.902 |
| Linaclotide use (yes vs no) | -0.981289 | 0.331524 | 0.375 | 0.196–0.718 | 0.003 |

ηbaseline = -2.057672 +0.004759×age -0.212916×sexMale -0.618411×inpatientInpatient +0.263066×previous_colonoscopyYes +0.124892×family_historyYes -0.331521×hypertensionYes +2.359884×diabetesYes +1.808955×constipationYes

ηextended = -1.522999 +0.002469×age -0.258267×sexMale -0.637191×inpatientInpatient +0.228439×previous_colonoscopyYes +0.085842×family_historyYes -0.341115×hypertensionYes +2.370335×diabetesYes +1.832622×constipationYes +0.372605×mosaprideYes -0.777685×prep_timeSplit-dose +0.017202×prep_agentPEG-ES -0.981289×linaclotideYes

For both models, predicted probability was calculated as p = exp(η)/[1 + exp(η)]. Each categorical term equals 1 for the named category and 0 for its stated reference category. Coefficients shown are from the final models fitted to all 2,283 participants and are reported to six decimal places to permit reproduction of individual predictions. Bootstrap resampling was used to assess internal performance and did not replace these final fitted coefficients.

**Abbreviations:** β, logistic regression coefficient; SE, standard error; OR, odds ratio; CI, confidence interval; PEG-ES, polyethylene glycol-electrolyte solution.

**Supplementary Table S2. Variance inflation factors for the baseline and extended prediction models**

| Model | Predictor term | VIF |
| --- | --- | --- |
| Baseline | age | 1.063 |
| Baseline | sexMale | 1.036 |
| Baseline | inpatientInpatient | 1.053 |
| Baseline | previous_colonoscopyYes | 1.047 |
| Baseline | family_historyYes | 1.008 |
| Baseline | hypertensionYes | 1.035 |
| Baseline | diabetesYes | 1.109 |
| Baseline | constipationYes | 1.023 |
| Extended | age | 1.084 |
| Extended | sexMale | 1.046 |
| Extended | inpatientInpatient | 1.236 |
| Extended | previous_colonoscopyYes | 1.053 |
| Extended | family_historyYes | 1.013 |
| Extended | hypertensionYes | 1.036 |
| Extended | diabetesYes | 1.125 |
| Extended | constipationYes | 1.030 |
| Extended | mosaprideYes | 1.023 |
| Extended | prep_timeSplit-dose | 1.019 |
| Extended | prep_agentPEG-ES | 1.281 |
| Extended | linaclotideYes | 1.037 |

All predictors had one degree of freedom. VIF values <5 were considered to indicate the absence of problematic multicollinearity. The maximum VIF in the extended model was 1.281.

**Supplementary Table S3. Formal segment-by-predictor interaction tests**

| Predictor | Wald χ² | df | Unadjusted P value | BH-adjusted P value |
| --- | --- | --- | --- | --- |
| Age | 2.850 | 2 | 0.241 | 0.421 |
| Sex | 2.027 | 2 | 0.363 | 0.421 |
| Previous colonoscopy | 2.632 | 2 | 0.268 | 0.421 |
| Diabetes mellitus | 44.137 | 2 | <0.001 | <0.001 |
| Constipation | 1.998 | 2 | 0.368 | 0.421 |
| Preparation timing | 2.982 | 2 | 0.225 | 0.421 |
| Preparation agent | 8.216 | 2 | 0.016 | 0.066 |
| Linaclotide use | 0.542 | 2 | 0.762 | 0.762 |

Interactions were estimated in long-format logistic models containing segment, the predictor, the segment-by-predictor interaction, and the remaining covariates from the segment-specific models. Patient-cluster robust covariance estimates accounted for the three segment outcomes contributed by each participant. P values were adjusted across the eight interaction tests using the Benjamini–Hochberg method. Within-segment estimates in Table 4 remain exploratory.

**Supplementary Table S4. Exploratory analyses of adenoma detection according to prediction model risk groups and bowel preparation quality**

| Analysis | Comparison | Adenoma events / Total (%) | OR (95% CI) | P value | Interpretation |
| --- | --- | --- | --- | --- | --- |
| Adenoma detection by predicted-risk group | Low | 190/910 (20.9%) |  |  | 95% CI for adenoma rate: 18.3%-23.7% |
| Adenoma detection by predicted-risk group | Intermediate | 157/969 (16.2%) |  |  | 95% CI for adenoma rate: 13.9%-18.7% |
| Adenoma detection by predicted-risk group | High | 79/404 (19.6%) |  |  | 95% CI for adenoma rate: 15.8%-23.8% |
| Adenoma logistic regression | Intermediate vs Low |  | 0.733 (0.580-0.926) | 0.009 | Unadjusted logistic regression; Low-risk group as reference |
| Adenoma logistic regression | High vs Low |  | 0.921 (0.687-1.235) | 0.583 | Unadjusted logistic regression; Low-risk group as reference |
| Adenoma logistic regression | Suboptimal vs Optimal |  | 0.712 (0.532-0.953) | 0.023 | Adjusted for age and sex |

**Abbreviations:** OR, odds ratio; CI, confidence interval. Predicted-risk groups were defined according to the model-predicted probability of suboptimal bowel preparation: low risk (<10%), intermediate risk (10–25%), and high risk (≥25%). Odds ratios for adenoma detection were calculated using the low-risk group as the reference. The association between bowel preparation quality and adenoma detection was adjusted for age and sex. This exploratory analysis was performed to evaluate whether the prediction model developed for suboptimal bowel preparation was associated with adenoma detection. Because adenoma detection was not the primary study outcome, these findings should be interpreted as exploratory.

**Supplementary Table S5. Exploratory analysis of the older comparison cohort**

**A. Characteristics according to bowel-preparation quality**

| Characteristic | Category | Overall (n = 3,146) | Optimal (n = 2,493) | Suboptimal (n = 653) | P value |
| --- | --- | --- | --- | --- | --- |
| Patients |  | 3146 | 2493 | 653 |  |
| Age, years |  | 59.6 ± 6.7 | 59.6 ± 6.7 | 59.6 ± 6.9 | 0.836 |
| Sex | Female | 1714 (54.5%) | 1355 (54.4%) | 359 (55.0%) | 0.775 |
|  | Male | 1432 (45.5%) | 1138 (45.6%) | 294 (45.0%) |  |
| Patient setting | Outpatient | 1242 (39.5%) | 933 (37.4%) | 309 (47.3%) | <0.001 |
|  | Inpatient | 1904 (60.5%) | 1560 (62.6%) | 344 (52.7%) |  |
| Anaesthesia | Anaesthesia | 2892 (91.9%) | 2296 (92.1%) | 596 (91.3%) | 0.490 |
|  | No anaesthesia | 254 (8.1%) | 197 (7.9%) | 57 (8.7%) |  |
| Family history of gastrointestinal malignancy | No | 2882 (91.6%) | 2302 (92.3%) | 580 (88.8%) | 0.004 |
|  | Yes | 264 (8.4%) | 191 (7.7%) | 73 (11.2%) |  |
| Previous colonoscopy | No | 2622 (83.3%) | 2096 (84.1%) | 526 (80.6%) | 0.031 |
|  | Yes | 524 (16.7%) | 397 (15.9%) | 127 (19.4%) |  |
| Polyp/adenoma | No | 2369 (75.3%) | 1872 (75.1%) | 497 (76.1%) | 0.591 |
|  | Yes | 777 (24.7%) | 621 (24.9%) | 156 (23.9%) |  |
| Hypertension | No | 1878 (59.7%) | 1533 (61.5%) | 345 (52.8%) | <0.001 |
|  | Yes | 1268 (40.3%) | 960 (38.5%) | 308 (47.2%) |  |
| Diabetes mellitus | No | 2589 (82.3%) | 2140 (85.8%) | 449 (68.8%) | <0.001 |
|  | Yes | 557 (17.7%) | 353 (14.2%) | 204 (31.2%) |  |
| Constipation | No | 2515 (79.9%) | 2130 (85.4%) | 385 (59.0%) | <0.001 |
|  | Yes | 631 (20.1%) | 363 (14.6%) | 268 (41.0%) |  |
| Preparation timing | Same-day | 1306 (41.5%) | 990 (39.7%) | 316 (48.4%) | <0.001 |
|  | Split-dose | 1840 (58.5%) | 1503 (60.3%) | 337 (51.6%) |  |
| Preparation agent | Mannitol | 1023 (32.5%) | 801 (32.1%) | 222 (34.0%) | 0.365 |
|  | PEG-ES | 2123 (67.5%) | 1692 (67.9%) | 431 (66.0%) |  |
| Mosapride during preparation | No | 2974 (94.5%) | 2410 (96.7%) | 564 (86.4%) | <0.001 |
|  | Yes | 172 (5.5%) | 83 (3.3%) | 89 (13.6%) |  |
| Linaclotide during preparation | No | 2895 (92.0%) | 2294 (92.0%) | 601 (92.0%) | 0.987 |
|  | Yes | 251 (8.0%) | 199 (8.0%) | 52 (8.0%) |  |
| Colonoscopy indication | Polyp surveillance | 649 (20.6%) | 509 (20.4%) | 140 (21.4%) | 0.783 |
|  | Family history | 417 (13.3%) | 325 (13.0%) | 92 (14.1%) |  |
|  | Symptomatic investigation | 1805 (57.4%) | 1441 (57.8%) | 364 (55.7%) |  |
|  | Screening | 275 (8.7%) | 218 (8.7%) | 57 (8.7%) |  |
| Total BBPS score |  | 6.1 ± 0.8 | 6.4 ± 0.6 | 4.9 ± 0.3 |  |

Values are mean ± s.d. or n (%). Continuous variables were compared using Welch two-sample t-tests; categorical variables were compared using Pearson chi-square or Fisher's exact tests, as appropriate. P values are two-sided, unadjusted, and exploratory. Total BBPS was not tested because it defines the outcome.

**B. Extended multivariable logistic regression model**

| Predictor | Adjusted OR | 95% CI | P value |
| --- | --- | --- | --- |
| Age, per year | 1.001 | 0.987–1.015 | 0.896 |
| Male sex (vs female) | 1.046 | 0.866–1.263 | 0.641 |
| Inpatient (vs outpatient) | 0.561 | 0.404–0.778 | <0.001 |
| Previous colonoscopy (yes vs no) | 1.134 | 0.887–1.449 | 0.315 |
| Family history of gastrointestinal malignancy (yes vs no) | 1.383 | 1.012–1.888 | 0.042 |
| Hypertension (yes vs no) | 1.089 | 0.893–1.329 | 0.400 |
| Diabetes mellitus (yes vs no) | 3.692 | 2.924–4.662 | <0.001 |
| Constipation (yes vs no) | 4.326 | 3.486–5.368 | <0.001 |
| Mosapride during preparation (yes vs no) | 4.594 | 3.262–6.470 | <0.001 |
| Split-dose preparation (vs same-day) | 0.618 | 0.511–0.748 | <0.001 |
| PEG-ES (vs mannitol) | 1.278 | 0.906–1.802 | 0.162 |
| Linaclotide during preparation (yes vs no) | 0.931 | 0.658–1.317 | 0.687 |

All listed predictors were entered simultaneously. No univariable P value screening was used. OR, odds ratio; CI, confidence interval; PEG-ES, polyethylene glycol-electrolyte solution.

**C. Additional assessment of anaesthesia and colonoscopy indication**

| Variable or comparison | Adjusted OR | 95% CI | P value |
| --- | --- | --- | --- |
| No anaesthesia (vs anaesthesia) | 1.108 | 0.786–1.561 | 0.559 |
| Indication: family history (vs polyp surveillance) | 1.062 | 0.767–1.470 | 0.718 |
| Indication: symptomatic investigation (vs polyp surveillance) | 0.940 | 0.740–1.195 | 0.615 |
| Indication: screening (vs polyp surveillance) | 0.992 | 0.678–1.450 | 0.965 |

The adjusted global likelihood-ratio P value for colonoscopy indication was 0.844. Adding anaesthesia and indication jointly to the extended model did not improve model fit (likelihood-ratio P = 0.887).

**D. Model performance and exploratory transportability**

| Analysis | AUC | Optimism-corrected AUC | Brier score | Optimism-corrected Brier | Calibration intercept | Calibration slope |
| --- | --- | --- | --- | --- | --- | --- |
| Baseline | 0.732 | 0.727 | 0.146 | 0.147 | — | — |
| Extended | 0.748 | 0.742 | 0.139 | 0.141 | — | — |
| Younger extended model applied to older cohort | 0.729 | — | 0.149 | — | -0.026 | 0.663 |

Older-cohort internal performance estimates used 1,000 bootstrap resamples. The younger-cohort model was applied without refitting. Its performance is exploratory because the cohorts had different analysis exclusions. AUC, area under the receiver operating characteristic curve.

**Supplementary Table S6. Exploratory age-group-by-predictor interaction analyses**

| Predictor | Age ≤50 years, adjusted OR (95% CI) | Age >50 years, adjusted OR (95% CI) | Interaction P value | BH-adjusted P value |
| --- | --- | --- | --- | --- |
| Male sex | 0.772 (0.604–0.986) | 1.038 (0.859–1.256) | 0.211 | 0.458 |
| Inpatient setting | 0.533 (0.391–0.727) | 0.563 (0.405–0.783) | 0.937 | 0.948 |
| Previous colonoscopy | 1.395 (0.944–2.061) | 1.131 (0.885–1.446) | 0.923 | 0.948 |
| Family history of gastrointestinal malignancy | 1.092 (0.764–1.560) | 1.382 (1.012–1.888) | 0.273 | 0.508 |
| Hypertension | 0.714 (0.401–1.271) | 1.084 (0.888–1.323) | 0.607 | 0.877 |
| Diabetes mellitus | 10.906 (7.441–15.985) | 3.712 (2.939–4.689) | <0.001 | <0.001 |
| Constipation | 6.305 (4.657–8.538) | 4.335 (3.493–5.381) | 0.206 | 0.458 |
| Mosapride use | 1.459 (0.873–2.439) | 4.554 (3.231–6.418) | <0.001 | <0.001 |
| Split-dose preparation | 0.462 (0.364–0.587) | 0.616 (0.509–0.746) | 0.080 | 0.258 |
| PEG-ES | 1.018 (0.773–1.341) | 1.274 (0.902–1.798) | 0.467 | 0.758 |
| Linaclotide use | 0.372 (0.194–0.713) | 0.929 (0.656–1.315) | 0.010 | 0.045 |
| No anaesthesia | 1.149 (0.767–1.721) | 1.108 (0.786–1.561) | 0.943 | 0.948 |
| Colonoscopy indication (global) | — | — | 0.948 | 0.948 |

Cohort-specific odds ratios were estimated from models containing the complete common covariate set. Interaction P values were obtained by adding one age-group-by-predictor term at a time to the pooled common-covariate model. The indication interaction is a three-degree-of-freedom global test, so a single odds ratio is not shown. P values were adjusted across 13 interaction tests using the Benjamini–Hochberg method. These comparisons are exploratory because the cohorts had different analysis exclusions.

**Supplementary Table S7. Calendar-time trends, calendar-adjusted sensitivity analyses, and internal temporal validation**

**A. Calendar-period distributions**

| Period | N | Suboptimal, n (%) | Split-dose, n (%) | PEG-ES, n (%) | Linaclotide, n (%) | Mosapride, n (%) |
| --- | --- | --- | --- | --- | --- | --- |
| 2017-2020 | 880 | 91 (10.3%) | 544 (61.8%) | 517 (58.8%) | 27 (3.1%) | 49 (5.6%) |
| 2021-2023 | 990 | 165 (16.7%) | 555 (56.1%) | 547 (55.3%) | 92 (9.3%) | 41 (4.1%) |
| 2024-2025 | 413 | 170 (41.2%) | 199 (48.2%) | 413 (100.0%) | 19 (4.6%) | 30 (7.3%) |

**B. Calendar-adjusted extended model**

| Predictor | Adjusted OR | 95% CI | P value |
| --- | --- | --- | --- |
| Age, per year | 1.002 | 0.985–1.019 | 0.814 |
| Male sex (vs female) | 0.763 | 0.595–0.978 | 0.033 |
| Inpatient (vs outpatient) | 0.346 | 0.244–0.490 | <0.001 |
| Previous colonoscopy (yes vs no) | 1.613 | 1.189–2.189 | 0.002 |
| Family history of gastrointestinal malignancy (yes vs no) | 1.079 | 0.751–1.551 | 0.680 |
| Hypertension (yes vs no) | 0.782 | 0.440–1.390 | 0.402 |
| Diabetes mellitus (yes vs no) | 6.174 | 4.109–9.277 | <0.001 |
| Constipation (yes vs no) | 5.163 | 3.781–7.050 | <0.001 |
| Mosapride use (yes vs no) | 1.438 | 0.846–2.443 | 0.179 |
| Split-dose preparation (vs same-day) | 0.483 | 0.379–0.617 | <0.001 |
| PEG-ES (vs mannitol) | 0.772 | 0.574–1.039 | 0.087 |
| Linaclotide use (yes vs no) | 0.372 | 0.194–0.716 | 0.003 |
| Calendar period: 2021–2023 (vs 2017–2020) | 1.636 | 1.221–2.191 | <0.001 |
| Calendar period: 2024–2025 (vs 2017–2020) | 5.370 | 3.546–8.132 | <0.001 |

**C. Contribution of calendar period to model fit**

| Model | Calendar-period LR P value | AUC without calendar period | AUC with calendar period |
| --- | --- | --- | --- |
| Baseline | <0.001 | 0.722 | 0.762 |
| Extended | <0.001 | 0.761 | 0.789 |

**D. Internal temporal validation**

| Model | Development N/events | Validation N/events | Observed risk | Mean predicted risk | AUC | Brier | Calibration intercept | Calibration slope |
| --- | --- | --- | --- | --- | --- | --- | --- | --- |
| Baseline | 1559/224 | 724/202 | 27.9% | 22.9% | 0.767 | 0.155 | 0.368 | 0.888 |
| Extended | 1559/224 | 724/202 | 27.9% | 22.7% | 0.769 | 0.157 | 0.389 | 0.830 |

Calendar periods were used for descriptive and sensitivity analyses. Calendar-period likelihood-ratio tests compare each original model with the same model plus calendar period. Internal temporal validation fitted each model in 2017–2022 and applied it without refitting in 2023–2025. Ideal calibration intercept and slope values are 0 and 1, respectively. This was an internal temporal assessment, not external validation.

**Supplementary Table S8. Outcome-related sensitivity analysis incorporating severe preparation failures**

**Panel A. Cohort composition for sensitivity analysis**

| Cohort description | n | Composite outcome events | Event rate |
| --- | --- | --- | --- |
| Original BBPS-defined primary cohort | 2,283 | 426 | 18.7% |
| Incomplete colonoscopy due to poor preparation (otherwise eligible) | 42 | 42 | 100% |
| Examinations requiring rescue cleansing (otherwise eligible) | 90 | 90 | 100% |
| Expanded sensitivity-analysis cohort | 2,415 | 558 | 23.1% |

**Panel B. Performance of models refitted on the expanded sensitivity-analysis cohort**

| Metric | Baseline model | Extended model |
| --- | --- | --- |
| Apparent AUC | 0.704 | 0.747 |
| Optimism-corrected AUC | 0.691 | 0.740 |
| Apparent Brier score | 0.146 | 0.142 |
| Optimism-corrected Brier score | 0.147 | 0.143 |
| Optimism-corrected calibration intercept | −0.027 | −0.031 |
| Optimism-corrected calibration slope | 0.975 | 0.971 |

**Panel C. Performance of original primary-cohort models applied without refitting to the expanded cohort**

| Metric | Baseline model | Extended model |
| --- | --- | --- |
| AUC | 0.683 | 0.726 |
| Brier score | 0.147 | 0.144 |
| Calibration intercept | 0.152 | 0.125 |
| Calibration slope | 0.906 | 0.880 |

The composite adverse outcome combined the original BBPS-defined suboptimal bowel preparation, incomplete colonoscopy due to poor bowel preparation, and examinations receiving rescue cleansing. All severe preparation-failure cases were coded as outcome events. Bootstrap internal validation used 1,000 resamples. AUC, area under the receiver operating characteristic curve; BBPS, Boston Bowel Preparation Scale.
